# Supplementary material for: Dp71 and intellectual disability in Indonesian patients with Duchenne muscular dystrophy
Source: PLoS One. 2022 Oct 31;17(10):e0276640. doi: 10.1371/journal.pone.0276640 (PMC9621454; doi:10.1371/journal.pone.0276640)
Supplement: Attachment — Submitted filename: Response to reviewers_Kristy_rev.edited.docx [file pone.0276640.s002.docx]

**RESPONSES TO REVIEWER AND EDITOR:**

The editor and reviewer comments are in italics in the material below, and our responses are regular.

*Journal Requirements:*

*When submitting your revision, we need you to address these additional requirements.*

*1. Please ensure that your manuscript meets PLOS ONE's style requirements, including those for file naming. The PLOS ONE style templates can be found at*

[*https://journals.plos.org/plosone/s/file?id=wjVg/PLOSOne_formatting_sample_main_body.pdf*](https://journals.plos.org/plosone/s/file?id=wjVg/PLOSOne_formatting_sample_main_body.pdf)  *and*

[*https://journals.plos.org/plosone/s/file?id=ba62/PLOSOne_formatting_sample_title_authors_affiliations.pdf*](https://journals.plos.org/plosone/s/file?id=ba62/PLOSOne_formatting_sample_title_authors_affiliations.pdf)

Thank you, we have checked and met PLOS ONE’s style requirements and file naming.

*2. Please provide additional details regarding participant consent. In the ethics statement in the Methods and online submission information, please ensure that you have specified what type you obtained (for instance, written or verbal, and if verbal, how it was documented and witnessed). If your study included minors, state whether you obtained consent from parents or guardians. If the need for consent was waived by the ethics committee, please include this information.*

*Once you have amended this/these statement(s) in the Methods section of the manuscript, please add the same text to the “Ethics Statement” field of the submission form (via “Edit Submission”).*

*For additional information about PLOS ONE ethical requirements for human subjects research, please refer to* [*http://journals.plos.org/plosone/s/submission-guidelines#loc-human-subjects-research*](http://journals.plos.org/plosone/s/submission-guidelines#loc-human-subjects-research)

Thank you, we have provided details regarding participant consent in the Methods section:

“Patients older than 12 years old and/or their parents or guardians (for patients <12 years old) signed a written informed consent form to be included in this study. The Institutional Review Board of the Faculty of Medicine, Public Health and Nursing, Universitas Gadjah Mada/Dr. Sardjito General Hospital approved this study (KE/FK/0894/EC/2020 and KE/FK/0180/EC/2021).”

*3. Thank you for stating the following in the Funding Section of your manuscript:*

*“This work was supported by a grant from the Faculty of Medicine, Public Health and Nursing, Universitas Gadjah Mada, Indonesia and 3 billion, Inc, South Korea.”*

*Please note that funding information should not appear in the Acknowledgments section or other areas of your manuscript. We will only publish funding information present in the Funding Statement section of the online submission form.*

*Please remove any funding-related text from the manuscript and let us know how you would like to update your Funding Statement. Currently, your Funding Statement reads as follows:*

*"This research is funded by the Faculty of Medicine, Public Health and Nursing, Universitas Gadjah Mada, Indonesia and 3 billion, Inc, South Korea. The funders had no role in study design, data collection and analysis, decision to publish, or preparation of the manuscript.”*

We have omitted the funding-related text from the manuscript. We agree on the statement in the funding statement section of the online submission form.

*Please include your amended statements within your cover letter; we will change the online submission form on your behalf.*

We have included amended statements in the cover letter. Thank you for your kind help.

*Additional Editor Comments (if provided):*

*Both reviewers, who are expert in the field, have raised some criticism that authors have to address. In my opinion, the manuscript would furtherly benefit by adding a table detailing all mutations identified in DMD patients included in the study. This Table would help the reader in interpreting the impact of the variants on the different Dp isoforms.*

Thank you for your suggestion. We have added a table detailing all mutations identified in DMD patients included in the study (Table 1).

***Reviewers' comments:***

***Comments to the Author***

***Reviewer #1:***

*Duchenne muscular dystrophy (DMD) is an X-linked recessive neuromuscular disorder which causes progressive muscle weakness leading to loss of ambulation in the mid-adolescent years. Affected males generally present in the first few years of life with motor symptoms and enlarged calves. However, neurodevelopmental disorders are increasingly recognized features and can be the initial presenting symptoms. The Authors report the results of a cross-sectional study aiming to characterize cognitive and neurodevelopmental profiles in a cohort of 48 patients with molecular diagnosis of DMD, aged between 5 and 18 years. Cognitive function was measured by intelligence quotient testing using the Wechsler Intelligence Scale for Children, and adaptive function tests with Vineland Adaptive Behavior Scales. The Autism Mental Status Exam and Abbreviated Conner's Rating Scale were used to screen autism spectrum disorder (ASD) and attention deficit and hyperactivity disorder (ADHD), respectively.*

*They found that patients with DMD have in general an IQ score lower compared to their peers and present with intellectual disability in about 20% of cases. No case of ASD, and only two boys with ADHD are reported. When trying a genotype-phenotype correlation, they conclude that DMD patients with mutation affecting Dp71 isoform of dystrophin are at higher risk to develop intellectual disability, but not ASD or ADHD.*

*The study is not original, as the knowledge that mutations disrupting the dystrophin brain isoforms Dp140 and Dp71 are more frequently associated with lower IQ scores in DMD patients, is not new. However, some results, such as the absence of patients with ASD and the low rate of patients with ADHD are interesting as they differ from what has been reported so far.*

Thank you very much for these encouraging comments from this reviewer. We have emphasized our findings from previous studies in the Discussion section, particularly the absence of patients with ASD and the low rate of patients with ADHD compared to the previous result.

*Critical points*

*- In tables there is no mention of the number of patients included in each dystrophin disruption group (Dp427, Dp140 e Dp71);*

Thank you for your suggestions. We have added the number of patients included in each dystrophin disruption group in table 2 and table 3.

*- Furthermore, in the tables it should be better clarified the difference* *between which groups the significance is referred.*

We have clarified the difference between which groups the significance is referred to. We have added, "The Dp71 group significantly had the lowest cognitive function with a total IQ score of 46 ± 24.83 compared to dp427 and dp140 group (*p*=0.005 and 0.001, consecutively) accompanied with scores for verbal abilities of 49.75 ± 26.73 (*p*=0.013 and 0.005, consecutively), and for performance abilities of 45.25 ± 24.80 (*p*=0.001 and 0.002, consecutively)” at result section.

*- It would also be interesting to subdivide the patients within the same group according to the type of mutation - deletions vs duplications - and evaluate, if any, differences in these subgroups.*

We have subdivided the patient within the same group according to the type of mutation (deletion, duplication, and point mutation) and evaluated the difference in cognitive profile, the prevalence of intellectual disability, ADHD, and ASD between the group (Table 5).

- *In my opinion, the absence of patients with ASD and the low rate of patients with ADHD should be underlined and widely discussed as they differ markedly from what has been reported so far.*

Thank you very much for your opinion. We have underlined and added discussion about the absence of patients with ASD, and the low rate of patients with ADHD: “Correlation between types of mutation (deletion, duplication, and point mutation) and severity of symptoms (ambulatory and non-ambulatory) with cognitive profiles and prevalence of intellectual disability and ADHD in each dystrophin disruption group were also analyzed. No significant difference was found in the IQ mean and the prevalence of intellectual disability and ADHD in each type of mutation. At the same time, there is a significant correlation between mutation types and adaptive and communication functions in the Dp427 group. Previous studies also reported no significant difference in mean IQ in each type of mutation [23]. Furthermore, the significant difference in cognitive profiles, adaptive profiles, and the prevalence of intellectual disability and ADHD was not observed for the severity of symptoms in each group.”

***Reviewer #2:***

*The article is well done and the topic is very interesting.*

*Anyway, data presented, even of interesting, appear partially in line with what available in literature. This could be due to a too small cohort or due to the application of evaluation methods not as sensitive as needed to analyze diseases object of investigation.*

*As said by the Author themselves, infact, the "Autism mental status exam" used by them, has a sensitivity of 81.2%.*

*Therefore, it could have been used a different diagnostic methods, more sensible and specific than previous (i.e., ADI-ADOS-2).*

Thank you very much for your kind comments. We have added “Other different diagnostic methods that are more sensible and more specific could have been used (i.e., ADI-ADOS-2)” in the Discussion section: “Other different diagnostic methods that are more sensible and more specific could have been used (i.e., ADI-ADOS-2).”

*Another diagnostic method could also be used for the diagnosis of attention deficit disorder (Nepsy II in its dedicated subparts).*

We have added “Another instrument to diagnose ADHD also available, for example Nepsy II in its dedicated subparts” in the Discussion section.

*Moreover, since the age of the sample is very large and Duchenne muscular dystrophy is a progressive pathology, it could also be useful to differentiate the cohort according to the level of severity of symptoms (for example ambulatory and non-ambulatory).*

We added a table differentiating the cohort according to the severity of symptoms. There is no difference in IQ, ASD, and ADHD according to the severity of symptoms (Table 6).

*Concerning IQ score much lower in dp71 group, this data is partially confirmed in literature but, in my opinion, this group should be enlarge to provide a wider benchmark and compare it.*

As suggested by the reviewer, we have already added more patients to this study. The new Table has been inserted in the text accordingly. Unfortunately, there were no additional patients with Dp71 mutation.

*For these findings, the paper need major work with a further evaluation for publication*

We have added additional DMD patients for further analysis and expanded the Discussion section.
