# Supplementary material for: Dp71 and intellectual disability in Indonesian patients with Duchenne muscular dystrophy
Source: PLoS One. 2022 Oct 31;17(10):e0276640. doi: 10.1371/journal.pone.0276640 (PMC9621454; doi:10.1371/journal.pone.0276640)
Supplement: Attachment — Submitted filename: Responses to reviewer and editor_rev2.docx [file pone.0276640.s003.docx]

**RESPONSES TO REVIEWER AND EDITOR:**

The editor and reviewer comments are in italics in the material below, and our responses are regular.

***Journal Requirements:***

*Please review your reference list to ensure that it is complete and correct. If you have cited papers that have been retracted, please include the rationale for doing so in the manuscript text, or remove these references and replace them with relevant current references. Any changes to the reference list should be mentioned in the rebuttal letter that accompanies your revised manuscript. If you need to cite a retracted article, indicate the article’s retracted status in the References list and also include a citation and full reference for the retraction notice.*

Our manuscript has not cited papers that have been retracted.

***Additional Editor Comments (if provided):***

*Concerning table 1, I believe the identified mutations should be reported according to the HGVS nomenclature. It's useful indicate the interval of exons deleted or duplicated but authors should complete table also reporting the expected phenotype DMD/BMD. Similarly, the indication "point mutation" for some patients seems very incomplete. These changes and that suggested by reviewers are mandatory.*

Thank you for your suggestions. We have already specified the mutation detail as for point mutation according to HGVS nomenclature. Moreover, we have excluded three patients with the expected BMD phenotype as per reviewer #1 suggestion, so all patients have DMD phenotype.

***Reviewers' comments:***

***Review Comments to the Author***

*Reviewer #1: The present version of the manuscript is much improved compared to the previous one. The Authors adressed all my suggestions.*

*I still have a few comments:*

*- Table 1. In the deletion group Dp427, the deletion 3-7 (patient NRR, 15 years) is listed: this is reported among the Malhotra’s exceptions to the reading frame rule. In fact, it is associated to a BMD severe phenotype.*

*Similarly in the deletion group Dp140, the deletions 48-49 (patient MA, 8 years) and 45-49 (patient AS, 11 years) are listed. Both these deletions are in frame and usually result in a BMD phenotype. These three patients must therefore be eliminated from the study.*

Thank you for your comments. We have already excluded the deletions 3-7 (patient NRR, 15 years) from the deletion group Dp427 also the deletions 48-49 (patient MA, 8 years) and 45-49 (patient AS, 11 years) from the deletion group Dp140.

*- Table 2. The Measure Unit for CK and CKMB should be indicated;*

Thank you for your suggestion. We have already added the measuring unit for CK (U/L) and CKMB (U/L) in Table 2.

*- Table 3. WISC group. Specify better that the significance of “p” is between the Dp71 group and the other two groups, as I understand or not;*

Thank you for your suggestions. We have already added the following sentences: “The Dp71 group significantly had the lowest cognitive function with total IQ score of 46.0 ± 24.8 compared to the other two groups (*p*=0.003) accompanied with scores for verbal abilities of 49.75 ± 26.73 (*p*=0.009), and for performance abilities of 45.3 ± 24.8 (*p*=0.002).”

*- Table 4. Specify better the significance for the intellectual disability to which group it refers;*

Thank you for your suggestions. We have already added the following sentences: “The incidence of intellectual disability differed significantly between the Dp71 group and the other two groups (*p*=0.009). All patients within the Dp71 group had intellectual disabilities.”

*- pag.15, line 17. I would say “motor” skills rather than “motoric” skills.*

Thank you for your suggestion. We have already replaced “motoric” skills with “motor” skills.
